# Domain-Aware Interpretable Machine Learning Model for Predicting Postoperative Hospital Length of Stay from Perioperative Data: A Retrospective Observational Cohort Study

**DOI:** 10.3390/bioengineering13020147

**Published:** 2026-01-27

**Authors:** Iqram Hussain, Joseph R. Scarpa, Richard Boyer

**Affiliations:** 1 Department of Anesthesiology, Weill Cornell Medicine, Cornell University, New York, NY 10065, USA; jos9335@med.cornell.edu (J.R.S.); rbb9007@med.cornell.edu (R.B.); 2 Cornell Tech, New York, NY 10044, USA

**Keywords:** postoperative length of stay (LOS), perioperative medicine, surgical outcomes, interpretable machine learning, domain-aware modeling

## Abstract

Background and Objective: Postoperative hospital length of stay (LOS) reflects surgical recovery and resource demand but remains difficult to predict due to heterogeneous perioperative trajectories. We aimed to develop and validate an interpretable machine learning framework that integrates multimodal perioperative data to accurately predict LOS and uncover clinically meaningful drivers of prolonged hospitalization. Methods: We studied 97,937 adult surgical cases from a large perioperative registry. Routinely collected perioperative data included patient demographics, comorbid conditions, preoperative laboratory values, intraoperative physiologic summaries, and procedural characteristics. Length of stay was modeled using a supervised regression approach with internal cross-validation and independent holdout evaluation. Model performance was assessed at both the cohort and individual levels, and explanatory analyses were performed to quantify the contribution of clinically defined perioperative domains. Results: The model achieved R^2^ = 0.61 and MAE ≈ 1.34 days on the holdout set, with nearly identical cross-validation performance (R^2^ = 0.60, MAE ≈ 1.34 days). Operative duration, diagnostic complexity, intraoperative hemodynamic variability, and preoperative laboratory indices—particularly albumin and hematocrit—emerged as the strongest determinants of postoperative stay. Patients with shorter recoveries typically had brief operations, stable physiology, and normal laboratory profiles, whereas prolonged hospitalization was linked to complex procedures, malignant or respiratory diagnoses, and lower albumin levels. Conclusions: Interpretable machine learning enables accurate and generalizable estimation of postoperative LOS while revealing clinically actionable perioperative domains. Such frameworks may facilitate more efficient perioperative planning, improved allocation of hospital resources, and personalized recovery strategies.

## 1. Introduction

Postoperative hospital length of stay (LOS) is a key indicator of surgical recovery, hospital efficiency, and perioperative quality of care [1,2]. Prolonged LOS increases healthcare costs, exposes patients to additional complications, and often reflects delayed recovery driven by modifiable preoperative or intraoperative factors [3]. Accurate prediction of LOS before or during surgery can therefore facilitate proactive discharge planning, optimize operating room scheduling, and enable targeted interventions for patients at elevated risk of prolonged hospitalization [4,5].

Extended hospital stay is also closely linked to adverse postoperative outcomes, including complications, readmissions, and mortality [6]. Patients with longer recoveries face higher rates of infection, thromboembolic events, and functional decline, making LOS a pragmatic surrogate marker of perioperative recovery and hospital performance [7]. Enhancing the accuracy and interpretability of LOS prediction can help clinicians identify vulnerable patients early and guide data-informed perioperative management strategies.

Traditional tools for predicting postoperative LOS have typically relied on a limited set of preoperative variables, linear regression models, or procedure-specific scoring systems [8,9,10]. Such approaches often fail to capture the complex interplay between physiological, procedural, and biochemical factors that influence recovery. Moreover, conventional models provide minimal interpretability, limiting clinical trust and hindering integration into perioperative decision-support workflows [11,12].

Recent advances in artificial intelligence (AI) and digital health technologies have transformed modern healthcare by enabling data-driven preoperative risk stratification, personalized care, and optimization of healthcare resources across diverse clinical settings [13,14,15,16,17,18]. In particular, machine learning (ML) approaches have demonstrated substantial potential in the management of chronic diseases, perioperative risk assessment, and prediction of hospitalization-related outcomes, including LOS, readmission, and postoperative recovery trajectories [19,20,21,22,23]. The growing availability of large-scale, multimodal perioperative datasets—integrating static clinical characteristics with high-resolution intraoperative physiological signals—has further accelerated the adoption of ML-based predictive models in perioperative medicine.

However, the clinical translation of these models has been limited by concerns regarding transparency, interpretability, and trust. Recent advances in interpretable ML have enhanced explainability and clinical trust in the analysis of large-scale, multimodal perioperative datasets that integrate static clinical variables with dynamic intraoperative signals [24,25,26,27]. To address the interpretability, explainable artificial intelligence (XAI) techniques—including feature attribution methods [12,28,29] and global explanation approaches [30,31,32,33]—are employed. In perioperative medicine, where clinical accountability and interpretability are paramount, understanding why a model predicts prolonged LOS is as important as achieving high predictive accuracy.

In this context, domain-aware modeling plays a crucial role by structuring diverse perioperative variables into clinically coherent domains, where each domain represents a meaningful aspect of the surgical journey—for example, preoperative laboratories, intraoperative physiology, diagnoses, procedures, and durations. By grouping related variables into these interpretable clinical domains, the model captures higher-order relationships within and across categories that would be obscured in feature-level analyses. This organization enhances interpretability how much each clinical domain contributes to model performance, enabling both transparent interpretation and targeted understanding of the perioperative factors that most strongly influence postoperative LOS. The key contributions of this study are threefold.

- First, we present an interpretable, domain-aware machine-learning framework that leverages routinely collected perioperative data—including patient characteristics, laboratory results, physiologic measures, and procedural information—to prospectively estimate postoperative length of stay.
- Second, the model demonstrates robust and generalizable performance while identifying clinically meaningful determinants of prolonged hospitalization, such as operative duration, diagnostic complexity, and perioperative physiologic and biochemical perturbations.
- Third, by integrating feature-level attribution with domain-level analysis, the framework provides interpretable, clinically grounded insight into how distinct perioperative data streams jointly shape postoperative recovery and hospital resource utilization.

## 2. Materials and Methods

**Figure 1:**
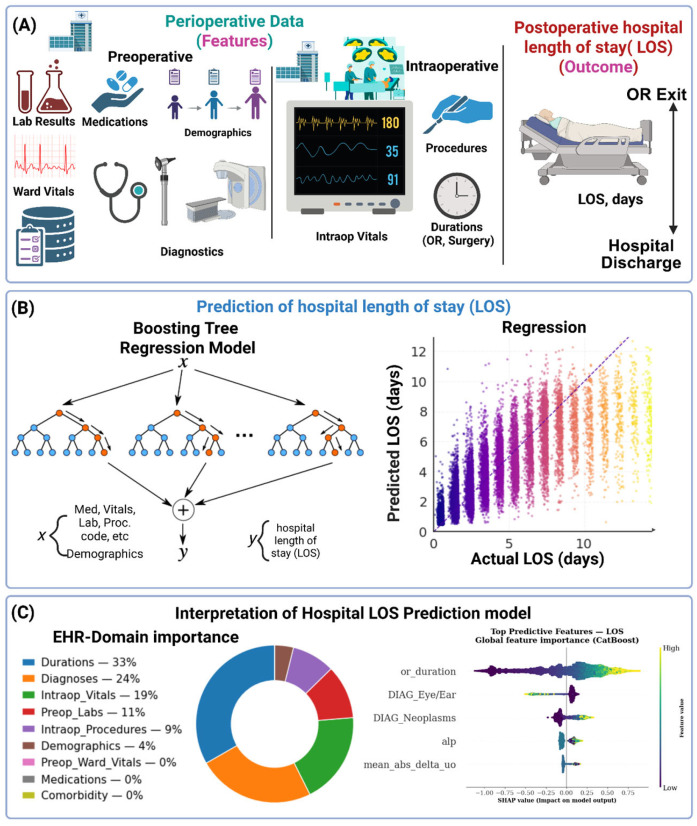
Predictive framework and interpretability of hospital length of stay (LOS, days). (**A**) Perioperative features integrated for LOS prediction. (**B**) Gradient-boosted tree regression model with observed versus predicted postoperative LOS (days). (**C**) Domain-level SHAP importance and global feature contributions, highlighting durations, diagnoses, and intraoperative vitals as key determinants of prolonged hospitalization.

This study presents a domain-aware, interpretable machine learning framework for predicting postoperative hospital LOS using routinely collected perioperative data (Figure 1). The analytic pipeline integrates structured features spanning preoperative, intraoperative, and postoperative phases—including demographics, comorbidities, laboratory results, physiologic time-series summaries, and procedural information. A gradient-boosted decision-tree model was trained to estimate postoperative LOS, with transparency achieved through clinically structured, domain-level feature grouping and complementary interpretability analyses.

### 2.1. Data Sources

This retrospective study analyzed perioperative and laboratory data sourced from the INSPIRE database, comprising operative cases from Seoul National University Hospital between 2011 and 2020 [34,35]. Adult, non-obstetric surgical encounters with complete perioperative timestamps and structured preoperative, intraoperative, and ward variables were included. Emergency surgeries and cesarean sections were excluded to maintain a homogeneous elective surgical cohort. Because INSPIRE is fully de-identified and publicly accessible, this study was exempt from institutional review board oversight.

### 2.2. Cohort Characteristics

Adult, non-obstetric surgical encounters were selected from the INSPIRE perioperative dataset. Eligible cases included patients aged ≥ 18 years who underwent elective surgery with complete perioperative timestamps and available structured demographic, laboratory, intraoperative physiologic, diagnostic, and procedural data. Emergency surgeries and obstetric procedures were excluded. Encounters with missing timestamps, implausible or non-positive length of stay, or evidence of temporal data leakage were removed.

**Figure 2:**
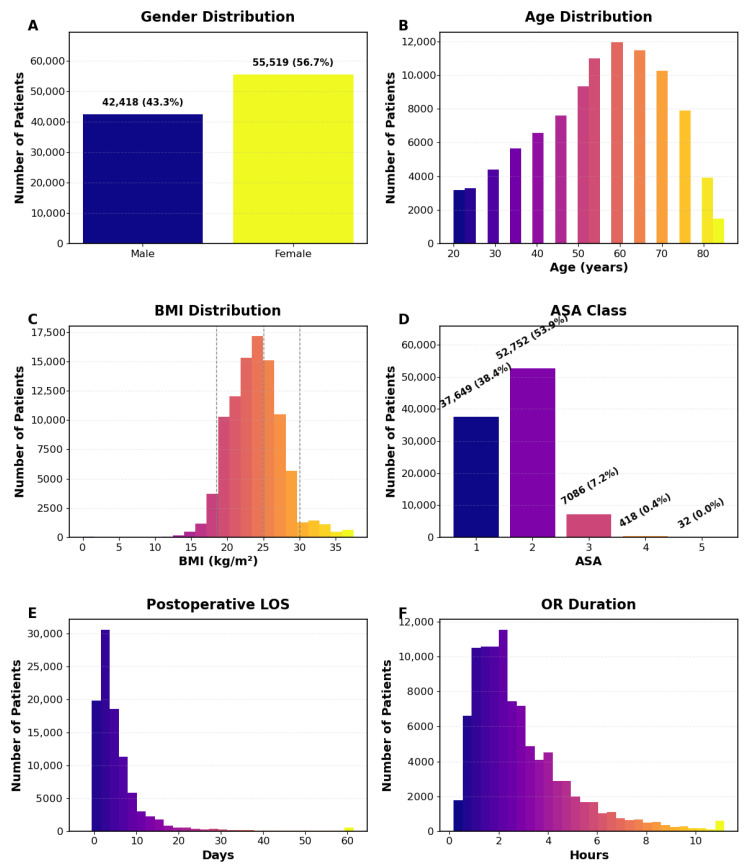
Cohort demographics and perioperative characteristics. Distribution of gender (**A**), age (**B**), BMI (**C**), ASA class (**D**), postoperative LOS (days) (**E**), and operative duration (hours) (**F**) for the study population, illustrating the heterogeneity of baseline and surgical factors incorporated in model development.

The final cohort included 97,937 surgical cases (Table 1, Figure 2). Median age was 55 years (IQR 45–65), with 56.7% male and 43.3% female; median BMI was 23.8 kg/m^2^ (IQR 21.5–26.0). Most patients were ASA-PS II (53.9%) or I (38.4%). General anesthesia was most common (81.5%), followed by neuraxial (9.3%) and monitored anesthesia care (9.1%). Major departments included general surgery (29.1%), orthopedics (12.1%), and otorhinolaryngology (11.0%); 7.0% were emergency cases. Median OR duration was 135 min (IQR 90–220), and anesthesia duration was 120 min (IQR 75–200). Preoperative labs were within normal ranges (albumin 4.1 g/dL, hematocrit 39.1%, creatinine 0.8 mg/dL).

### 2.3. Outcome Definition

The primary outcome was postoperative hospital LOS, measured in days. The LOS start time was defined as the operating-room exit time, defaulting to operation or anesthesia end time when unavailable. The end time corresponded to hospital discharge or, in cases of in-hospital death, the recorded time of death. Encounters with LOS ≤ 0 were excluded. To remove implausible values, LOS was capped at 90 days and further truncated at the 95th percentile. These thresholds were applied to limit the influence of extreme outliers, which represent a small fraction of cases and can disproportionately affect loss optimization in tree-based models. Clinically, very prolonged hospitalizations are often driven by rare events or non-clinical factors, such as discharge disposition delays or social barriers, that are not well captured by perioperative features. The resulting distribution showed a right-skewed pattern typical of perioperative recovery, with a median 3.6 days (IQR 1.6–6.6) and mean ± SD 5.7 ± 7.4 days (Figure 2E).

### 2.4. Perioperative Variables and Clinically Structured Domain Grouping

Predictors were derived from routinely collected perioperative data, restricted to variables available before or during surgery to prevent post-outcome leakage. The analytic feature set encompassed demographics (age, gender, BMI, ASA-PS), preoperative laboratory indices (albumin, chloride, hematocrit, CRP, and others), preoperative ward vital signs summarized by mean, minimum, maximum, and standard deviation (heart rate, respiratory rate, blood pressure), and intraoperative physiological measures expressed as means, ranges, and absolute deltas for key parameters including arterial and non-invasive blood pressure, heart rate, end-tidal CO_2_, tidal volume, minute ventilation, and urine output (Table 2). Procedural information was represented using one-hot NHSN-style categories such as breast, gastric, and hip-prosthesis surgery, while diagnostic information was reduced to binary indicators derived from ICD-10 chapters or clinically coherent diagnostic groups. Additional predictors included Charlson-style comorbidity flags and intraoperative duration metrics (anesthesia and operating-room time). Variables with poor standardization or potential temporal leakage—such as postoperative FiO_2_ changes, estimated blood loss, ECMO/CRRT indicators, or mortality timestamps—were excluded.

Each remaining feature was mapped to a human-readable domain label (e.g., demographics, preoperative laboratories, intraoperative vitals, procedures, diagnoses), enabling interpretation of model behavior both at the individual-feature and aggregated-domain levels.

### 2.5. Data Preprocessing

A domain-aware preprocessing pipeline was applied to ensure data integrity and prevent information leakage. Postoperative LOS was log-transformed to correct skewness. Continuous variables were median-imputed and robust-scaled, categorical variables were mode-imputed and one-hot encoded, and near-constant features (variance < 0.01) were removed. The resulting standardized matrices were used as input for feature selection and model training.

### 2.6. Feature Selection with Clinical Priors

To reduce dimensionality while maintaining clinical interpretability, we combined linear sparsity, tree-based attribution, and domain diversity. First, LassoCV with five-fold cross-validation was applied to the preprocessed training matrix to identify sparse linear predictors [36]. Second, a CatBoost regressor (iterations = 100, learning rate = 0.05, depth = 4) was trained to compute Shapley additive explanations (SHAP) values, and features with high mean absolute SHAP values were prioritized [30,37,38]. To avoid over-reliance on any single domain, we aggregated SHAP importance within domains and force-included the most informative feature from each high-impact group. Clinically essential variables such as age and ASA were preserved regardless of statistical ranking. Finally, we capped the selection at no more than eight features per domain and 20 features in total, resulting in a compact, interpretable panel spanning demographics, preoperative labs, intraoperative vitals, intraoperative procedures, diagnoses, and surgical duration.

### 2.7. Model Training and Validation

The final feature set was used to train a gradient-boosted decision tree model implemented with CatBoost [39]. We specified 300 boosting iterations, a depth of 6, and a learning rate of 0.05, with random state fixed for reproducibility. Predictions were generated in log space and then exponentiated back to minutes. To evaluate generalizability, we applied a GroupKFold strategy to account for subject-level clustering [36]. Performance was assessed both on the independent hold-out test set and through out-of-fold predictions from five-fold cross-validation. Metrics included mean absolute error (MAE), root mean squared error (RMSE), mean absolute percentage error (MAPE), and the coefficient of determination (R^2^), reported in both log and unlogged LOS space. To quantify uncertainty, we computed 95% confidence intervals for R^2^ and MAE using 1000 paired bootstrap resamples of the test predictions.

### 2.8. Model Explainability and Domain Ablation

To enhance clinical interpretability, we applied SHAP to quantify the contribution of each predictor to model output [38,40]. Global feature importance was summarized as the mean absolute SHAP value across the training and test sets, and results were visualized using SHAP summary plots.

$${SHAP}_{i}=\varphi_{i}\left(F,x\right)=\sum_{S\subseteq F\setminus \left\{i\right\}}\frac{\left|S\right|!\left(\left|M\right|-\left|S\right|-1\right)!}{\left|M\right|!}\left[F\left(x_{S\cup \left\{i\right\}}\right)-F\left(x_{S}\right)\right]\;$$

where *M* is the set of all features, *x_S_* is a subset of features *S*, *|S|* represents the cardinality of set *S*, *SHAP_i_* is the SHAP value for feature *i*, and *φ_i_*(*F*,*x*) is the SHAP value function for feature *i*.

To better capture domain-level insights, feature-level SHAP values were aggregated by predefined groups (e.g., demographics, preoperative labs, intraoperative vitals, procedures), producing a domain-wise ranking of importance. In parallel, we performed leave-one-domain-out ablation experiments using an XGBoost regressor with GroupKFold cross-validation [41]. For each domain, we re-trained the model without its features and computed the change in R^2^ (ΔR^2^) relative to the full model. Domains with larger ΔR^2^ values were considered more critical for predictive performance. We compared domain-level SHAP scores with ΔR^2^ drops to cross-validate importance rankings.

## 3. Results

### 3.1. Model Performance and Generalization

**Figure 3:**
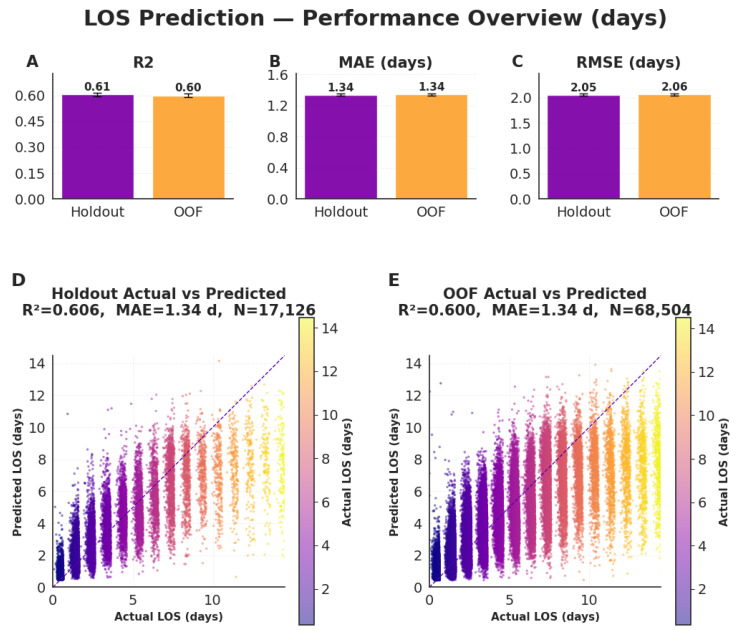
Model performance for postoperative hospital length of stay (LOS, days). Evaluation of regression performance across the independent holdout and out-of-fold (OOF) validation datasets. (**A**) Coefficient of determination (R^2^), (**B**) mean absolute error (MAE, days), and (**C**) root mean square error (RMSE, days) quantify predictive accuracy. (**D**,**E**) Scatter plots of predicted versus observed LOS (days) demonstrate strong calibration and consistent generalization across cohorts, with stable performance across the full postoperative LOS range.

The CatBoost regression model demonstrated consistent and robust performance in predicting postoperative hospital LOS (days) (Figure 3A–E). On the independent holdout dataset, the model achieved an R^2^ = 0.61, MAE = 1.34 days, and RMSE = 2.05 days. Performance on out-of-fold (OOF) cross-validation was nearly identical (R^2^ = 0.60, MAE = 1.34 days, RMSE = 2.06 days), underscoring the framework’s reproducibility and generalizability across cohorts.

Scatter plots of predicted versus observed LOS (Figure 3D–E) show strong calibration with tight clustering around the identity line, indicating reliable agreement between predicted and actual values across the full postoperative LOS range. Minor dispersion appears only among the longest-stay cases, reflecting natural variability in extended recovery durations. Overall, these results confirm that the proposed model accurately captures inter-patient variability in postoperative recovery time while maintaining stable generalization between holdout and cross-validation datasets.

### 3.2. Feature-Level Interpretability and Key Predictors

**Figure 4:**
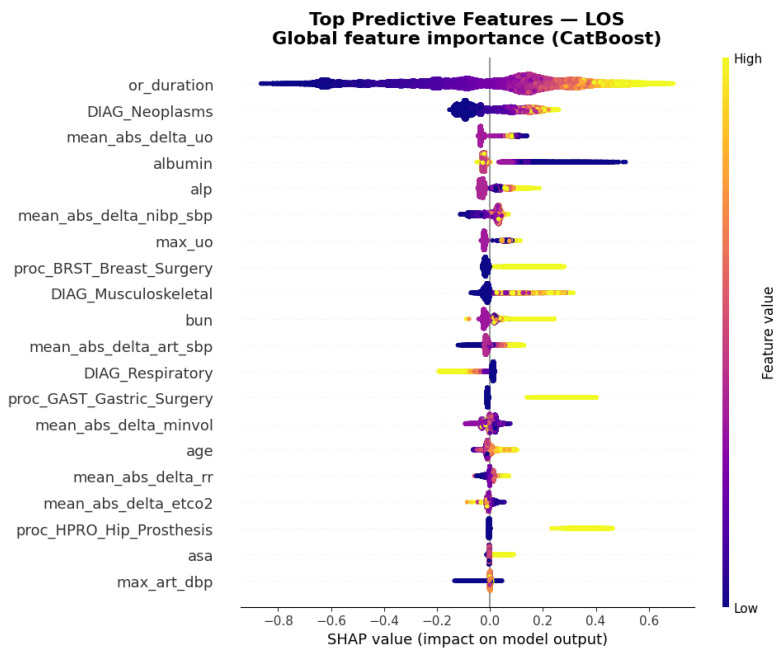
Global feature effects on postoperative LOS (days). TreeSHAP beeswarm for the final CatBoost model. Each point represents a patient; color encodes the feature value (low → high). Positive SHAP values indicate longer predicted LOS (days). Operative duration, diagnostic groups (e.g., neoplasms, respiratory), preoperative laboratories (albumin, chloride), intraoperative physiology (arterial pressure, EtCO_2_, respiratory rate, urine output dynamics), and procedure classes (breast, gastric, hip prosthesis) are among the leading contribution.

Global SHAP analysis identified operative duration as the most influential predictor of postoperative hospital LOS (days) (Figure 4). Diagnostic categories—particularly neoplasms, and respiratory diseases—also had strong positive contributions, indicating that higher feature values within these groups were consistently associated with longer hospital stays. Among laboratory indices, albumin and alkaline phosphatase (ALP) emerged as key biochemical correlates, with lower albumin and higher ALP levels linked to prolonged hospitalization. Dynamic intraoperative parameters—including mean absolute changes in urine output (ΔUO), arterial systolic pressure (ΔSBP), non-invasive blood pressure (ΔNIBP), and ventilation measures such as Δminute volume, Δrespiratory rate, and ΔEtCO_2_—further contributed to predictions, highlighting the importance of physiologic variability during surgery. Procedural categories such as breast, gastric, and hip prosthesis surgeries also ranked prominently, reflecting procedure-specific recovery trajectories. Collectively, these findings show how the model integrates operative duration, diagnostic complexity, preoperative biochemistry, and intraoperative dynamics to generate physiologically interpretable predictions of postoperative LOS.

### 3.3. Domain-Aware Interpretability and Hierarchical Insights

**Figure 5:**
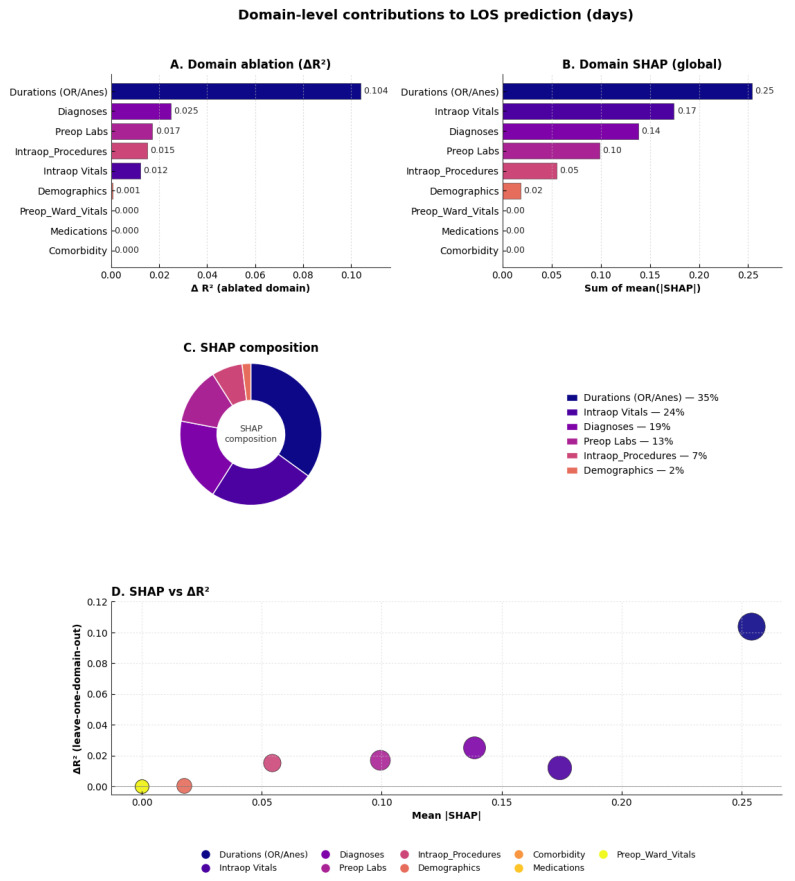
Domain-level contributions to postoperative hospital length of stay (LOS, days). (**A**) Leave-one-domain-out ablation showing the decline in explained variance (ΔR^2^) when each domain is excluded; durations (OR/anesthesia) and diagnoses produce the largest reductions. (**B**) Global SHAP importance aggregated by domain, confirming durations, diagnoses, intraoperative vitals, and preoperative labs as the dominant feature groups. (**C**) Donut chart of SHAP composition showing the proportional attribution of each domain (e.g., durations ≈ 33%, diagnoses ≈ 24%, intraoperative vitals ≈ 19%). (**D**) Mean |SHAP| versus ΔR^2^ bubble plot (bubble size ∝ SHAP magnitude), demonstrating agreement between interpretability and ablation analyses.

To capture higher-order structure across clinically related features, we implemented a domain-aware interpretability framework integrating leave-one-domain-out ablation and global SHAP aggregation (Figure 5A–D). Both complementary approaches revealed a consistent hierarchy of perioperative domains influencing LOS prediction.

Ablation testing showed that removing durations (OR/anesthesia) caused the largest performance drop (ΔR^2^ = 0.081), followed by diagnoses (ΔR^2^ = 0.032), intraoperative procedures (ΔR^2^ = 0.014), preoperative labs (ΔR^2^ = 0.012), and intraoperative vitals (ΔR^2^ = 0.008). Global SHAP aggregation confirmed these trends, ranking durations (0.25), diagnoses (0.20), intraoperative vitals (0.15), and preoperative labs (0.07) as the top domains. SHAP composition indicated proportional contributions of approximately 33%, 26%, 20%, and 10%, respectively.

Overall, these results emphasize that operative duration, diagnostic complexity, intraoperative physiologic variability, and preoperative laboratory status are the principal determinants of hospital LOS, while medications and ward vitals contributed minimally supporting the robustness and clinical interpretability of the domain-aware framework.

### 3.4. Multidomain Feature Dependencies Underlying LOS Prediction

**Figure 6:**
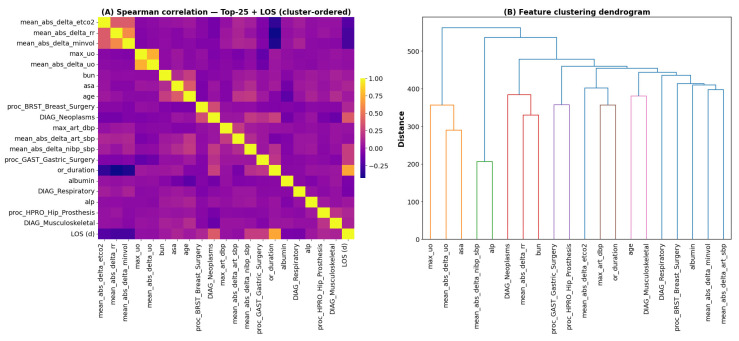
Cluster-ordered Spearman correlation matrix and hierarchical feature clustering. (**A**) Spearman correlations between the top 25 SHAP-selected predictors and postoperative length of stay (LOS), ordered using hierarchical Ward linkage to highlight correlated feature groups. (**B**) Corresponding dendrogram illustrating clinically coherent clusters spanning laboratory values, vital signs, diagnosis categories, demographic factors, and procedure-related features.

To characterize the structural relationships among the most influential predictors of postoperative length of stay (LOS), we quantified pairwise associations across the top 25 SHAP-ranked variables. The cluster-ordered Spearman correlation matrix (Figure 6A) reveals distinct blocks of covarying features that align with physiological, demographic, diagnostic, and procedural domains. Ward hierarchical clustering further resolves these dependencies, yielding clinically coherent groupings that reflect shared biological or perioperative mechanisms (Figure 6B). This multidomain organization underscores that LOS is governed not by isolated predictors but by coordinated patterns spanning multiple facets of patient status and surgical care.

### 3.5. Patient-Level and Personalized Interpretability

**Figure 7:**
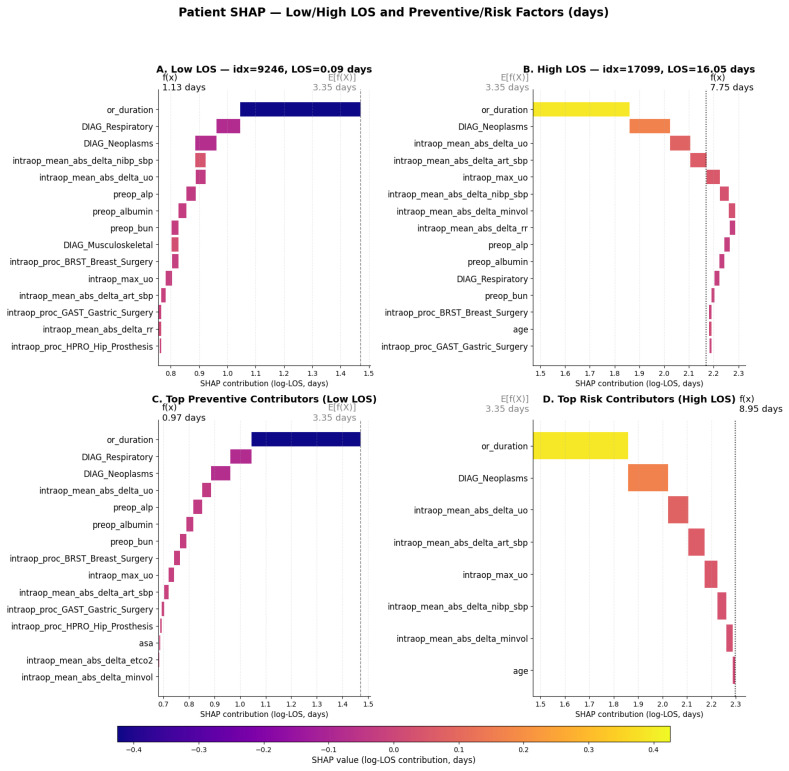
Patient-level SHAP explanations for postoperative LOS (days). (**A**) Example of a patient with short LOS (days), where brief operative duration and non-malignant diagnoses act as protective factors. (**B**) Example of a patient with prolonged LOS, driven by respiratory diagnosis, extended operative duration, and intraoperative instability. (**C**) Aggregated top preventive contributors across low-LOS cases. (**D**) Aggregated top risk contributors across high-LOS cases. These individualized and aggregate SHAP explanations illustrate the distinct perioperative patterns underlying short versus prolonged recovery trajectories.

Patient-specific SHAP waterfall plots illustrate how individual feature combinations influence LOS predictions (Figure 7A–D). For low-LOS cases, preventive contributors such as short operative duration, favorable diagnoses, stable intraoperative parameters, and normal preoperative labs were dominant (Figure 7A,C). Conversely, high-LOS cases were characterized by risk-enhancing drivers, including prolonged operative duration, intraoperative instability, and comorbid or malignant diagnoses (Figure 7B,D). These individualized SHAP explanations provide clinically intuitive narratives for both short and extended recoveries, offering potential utility for personalized perioperative planning and postoperative risk communication.

## 4. Discussion

We developed a domain-aware, interpretable machine-learning framework to predict postoperative hospital LOS using a large, heterogeneous perioperative cohort. By organizing perioperative data into clinically coherent domains—demographics, laboratories, intraoperative physiology, and procedures—the model achieved strong generalization across cohorts. This domain-aware structure enabled hierarchical interpretability, revealing key predictors at the feature, domain, and patient levels, and translating data-driven outputs into clinically meaningful insights for perioperative recovery.

### 4.1. Predictive Performance and Key Determinants of Hospital LOS

The model achieved consistent and robust predictive performance (R^2^ = 0.60), demonstrating strong calibration and generalization across both holdout and cross-validation cohorts. Operative duration emerged as the dominant determinant, reflecting its established role as a surrogate for surgical complexity, anesthetic exposure, and intraoperative resource utilization [42]. Diagnostic categories—particularly neoplasms, musculoskeletal, and respiratory disorders—were also highly influential, aligning with prior findings that link underlying disease burden and procedural type to delayed postoperative recovery [43,44]. Preoperative laboratory measures such as albumin, chloride, and hematocrit contributed significantly, consistent with biochemical markers of nutritional status, inflammation, and oxygen-carrying capacity known to affect surgical outcomes [45].

Moreover, intraoperative hemodynamic and ventilatory variability underscored the importance of physiologic stability during surgery, supporting evidence that fluctuations in arterial pressure and ventilation parameters are strong predictors of postoperative complications and extended hospitalization [46]. Although postoperative complications were not explicitly modeled to avoid temporal data leakage, these perioperative factors represent upstream determinants of complication risk and are therefore implicitly captured through their association with prolonged length of stay. Together, these results highlight the framework’s ability to integrate both procedural and dynamic physiologic signals to model recovery after surgery with clinical fidelity.

### 4.2. Interpretability and Clinical Relevance

By integrating feature attribution with domain-level ablation, the framework provided transparent, multi-scale interpretability. Both methods consistently identified durations, diagnoses, intraoperative vitals, and preoperative labs as the most influential domains shaping postoperative LOS [42,45,47,48]. At the feature level, operative duration, diagnostic complexity, and biochemical indices such as albumin and alkaline phosphatase emerged as dominant determinants, while intraoperative physiologic variability further modulated recovery patterns [43,44,45]. The alignment between feature- and domain-level analyses reinforces the robustness and clinical plausibility of the model’s explanations. At the patient level, individualized Shapley values narratives distinguished protective from risk-enhancing factors—linking short procedures, stable intraoperative physiology, and normal laboratory values with early discharge, and prolonged operations, hemodynamic instability, or adverse diagnoses with extended hospitalization. These interpretable relationships transform the model from a predictive algorithm into an explanatory, clinically grounded decision-support framework, strengthening confidence in ML-based perioperative risk assessment.

### 4.3. Comparison with Prior Work

Prior studies complement our findings by demonstrating the utility of machine-learning methods for predicting hospital resource utilization and improving operational efficiency [49,50]. For example, XGBoost can accurately predict cardiothoracic surgery duration and support data-driven capacity management by reducing delays in elective and acute surgical schedules [51]. Similarly, Light Gradient-Boosting Machine (LightGBM) models forecasts emergency department crowding and inform staffing strategies aligned with patient volume demand [52]. Gradient-boosting-based models—particularly XGBoost—achieved the highest predictive performance for postoperative length of stay (LOS), although other ensemble methods such as random forests demonstrated comparable performance [21,53]. Random forest models predicts postoperative LOS, intensive care unit admission, surgical bed utilization, and outpatient visit volumes in adult hospital populations [54]. In addition, operations research-based approaches, including integer linear programming and goal programming, have been shown to effectively optimize elective surgical scheduling and operating room utilization [20,53].

Most LOS prediction studies have often focused on narrow surgical cohorts, relied primarily on preoperative features, or lacked interpretable frameworks. Our work expands on these by demonstrating that large-scale multimodal perioperative data can be effectively harnessed in an interpretable boosting-tree framework. A key strength of the present study is the explicit use of electronic health record (EHR) domain-aware feature organization. By combining feature-level SHAP explanations with domain-level ablation analyses, we quantify not only which variables are influential, but also how entire perioperative domains—such as operative duration, diagnoses, intraoperative physiology, and preoperative laboratory status—contribute to LOS prediction. This hierarchical interpretability is particularly valuable in perioperative medicine, where data streams are inherently structured, and clinical decision-making often occurs at the EHR domain rather than individual-feature level.

Furthermore, by deliberately restricting predictors to preoperative and intraoperative data, our approach preserves temporal validity and positions LOS as a downstream outcome that implicitly reflects postoperative complications and recovery trajectories. The resulting model achieves strong and reproducible performance as an interpretable LOS decision-support tools for perioperative planning and hospital resource management.

### 4.4. Strength and Perspectives for Clinical Application

Key strengths of this study include the use of a large, real-world perioperative dataset, systematic feature engineering across pre-, intra-, and postoperative phases, and the integration of complementary interpretability techniques. By restricting predictors to preoperative and intraoperative information, the framework preserves clinical realism while avoiding postoperative data leakage, enabling predictions that are feasible within routine perioperative workflows. The integration of complementary interpretability techniques—feature-level SHAP attribution and domain-level ablation—provides multi-scale insight into the determinants of hospital length of stay, allowing clinicians to contextualize predictions within familiar perioperative domains rather than individual variables.

In current clinical practice, length of stay is calculated retrospectively from EHR timestamps and is therefore known only at discharge. The proposed framework shifts LOS assessment upstream by providing prospective estimates based on data available before or during surgery, enabling earlier and more informed perioperative planning. Our SHAP interpretability analyses further clarify which individual features and perioperative domains most strongly influence LOS predictions. Together, these capabilities support proactive discharge coordination, bed and operating room capacity management, and targeted allocation of postoperative resources.

### 4.5. Limitations and Future Directions

Limitations include potential unmeasured confounding (e.g., socioeconomic and hospital-level factors not captured in the dataset), the single-country setting which may limit generalizability, and residual noise in intraoperative signal summaries. Additionally, prediction error increased for extreme LOS outliers, suggesting that further work is needed to model rare, prolonged hospitalizations. Future studies should explore external validation across multi-institutional datasets, incorporation of additional perioperative variables such as postoperative complications and enhanced recovery protocol adherence, and integration with clinician-facing decision support tools. Deep learning models leveraging raw intraoperative waveform data may further improve accuracy, while hybrid approaches combining machine learning with mechanistic models could enhance interpretability. Ultimately, embedding interpretable LOS prediction into perioperative planning workflows may support proactive resource allocation, early discharge planning, and targeted interventions for high-risk patients.

## 5. Conclusions

Postoperative length of stay is a critical determinant of surgical outcomes, healthcare resource utilization, and patient recovery, yet remains difficult to predict accurately across heterogeneous surgical populations. In this large perioperative cohort of surgical cases, we developed an interpretable machine learning framework that achieved robust and generalizable prediction of postoperative hospital LOS. By combining feature-level interpretation with domain-level ablation, we identified convergent and clinically meaningful drivers of prolonged hospitalization. Case-level explanations further demonstrated how individual patient risk can be understood in a transparent manner. Collectively, these findings underscore the potential of interpretable machine learning to enhance perioperative decision-making, support resource allocation and discharge planning, and facilitate individualized risk stratification in clinical practice.

## Figures and Tables

**Table 1:** Baseline characteristics of the study cohort.

Characteristic	Value Format	Value
Cohort size (n)	Count	97,937
Gender	(distribution)	Male: 55,519 (56.7%); female: 42,418 (43.3%)
Race	(distribution)	Asian: 97,937 (100.0%)
ASA-PS	(distribution)	2.0: 52,752 (53.9%); 1.0: 37,649 (38.4%); 3.0: 7086 (7.2%); 4.0: 418 (0.4%); 5.0: 32 (0.0%)
Primary anesthetic type	(distribution)	General: 79,788 (81.5%); neuraxial: 9110 (9.3%); monitored anesthesia care: 8951 (9.1%); regional: 88 (0.1%)
Diagnostics department	(distribution)	General surgery: 28,548 (29.1%); orthopedics: 11,897 (12.1%); otorhinolaryngology: 10,784 (11.0%); OG: 10,579 (10.8%); OL: 9437 (9.6%); UR: 8641 (8.8%); NS: 7638 (7.8%); CTS: 6586 (6.7%); other: 3827 (3.9%)
Emergency case (n, %)	(n, %)	6848 (7.0%)
Age (y)	(Median [IQR]; Mean ± SD)	55.0 [45.0–65.0]; 54.7 ± 16.0
BMI (kg/m^2^)	(Median [IQR]; Mean ± SD)	23.8 [21.5–26.0]; 23.9 ± 3.7
OR duration (min)	(Median [IQR]; Mean ± SD)	135.0 [90.0–220.0]; 169.2 ± 116.8
Anesthesia duration (min)	(Median [IQR]; Mean ± SD)	120.0 [75.0–200.0]; 155.2 ± 114.1
Preop albumin (g/dL)	(Median [IQR]; Mean ± SD)	4.1 [3.9–4.3]; 4.0 ± 0.5
Preop chloride (mmol/L)	(Median [IQR]; Mean ± SD)	103.0 [102.0–106.0]; 103.7 ± 3.4
Preop hematocrit (%)	(Median [IQR]; Mean ± SD)	39.1 [35.2–42.0]; 38.7 ± 4.8
Preop hemoglobin (g/dL)	(Median [IQR]; Mean ± SD)	13.2 [11.9–14.2]; 12.9 ± 1.8
Preop creatinine (mg/dL)	(Median [IQR]; Mean ± SD)	0.8 [0.7–0.9]; 0.9 ± 0.7
Preop BUN (mg/dL)	(Median [IQR]; Mean ± SD)	13.0 [10.0–18.0]; 15.4 ± 8.0
Preop sodium (mmol/L)	(Median [IQR]; Mean ± SD)	139.0 [139.0–141.0]; 139.4 ± 2.6
Preop potassium (mmol/L)	(Median [IQR]; Mean ± SD)	4.0 [3.8–4.4]; 4.1 ± 0.4
Preop WBC (×10^9^/L)	(Median [IQR]; Mean ± SD)	6.3 [5.2–7.9]; 6.7 ± 2.4
Preop CRP (mg/L)	(Median [IQR]; Mean ± SD)	0.1 [0.0–0.5]; 1.0 ± 3.0
Preop fibrinogen (mg/dL)	(Median [IQR]; Mean ± SD)	301.0 [263.0–357.0]; 320.6 ± 84.5
Postoperative LOS (days)	(Median [IQR]; Mean ± SD)	3.57 [1.59–6.55]; 5.68 ± 7.36

Notes: ASA = American Society of Anesthesiologists physical status classification; BMI = body mass index; BUN = blood urea nitrogen; WBC = white blood cell count; CRP = C-reactive protein; OG = Obstetrics & Gynecology; OL = ophthalmology; UR = urology; NS = neurosurgery; CTS = cardiothoracic surgery.

**Table 2:** Description of features used in this study.

Feature Name	Description
or_duration	Total duration of surgery (minutes), representing operative time and procedural complexity.
DIAG_Neoplasms	Presence of neoplastic (cancer-related) diagnoses; reflects surgical oncology cases.
DIAG_Eye/Ear	Eye- and ear-related surgical diagnoses (e.g., ENT procedures).
mean_abs_delta_uo	Mean absolute change in intraoperative urine output across time; a marker of renal perfusion and volume status.
albumin	Preoperative serum albumin concentration (g/dL), reflecting nutritional and inflammatory status.
bun	Preoperative blood urea nitrogen (mg/dL); indicator of renal function and catabolic state.
proc_BRST_Breast_Surgery	Indicator variable for breast-related surgical procedures.
DIAG_Musculoskeletal	Musculoskeletal disease category (e.g., orthopedic surgery cases).
DIAG_Respiratory	Respiratory system-related diagnoses (e.g., thoracic or airway procedures).
mean_abs_delta_nibp_sbp	Mean absolute change in non-invasive systolic blood pressure; quantifies hemodynamic variability.
mean_abs_delta_art_sbp	Mean absolute change in invasive arterial systolic blood pressure; reflects intraoperative instability.
age	Patient age at the time of surgery (years).
proc_GAST_Gastric_Surgery	Indicator variable for gastric surgery (e.g., gastrectomy).
mean_abs_delta_minvol	Mean absolute change in minute ventilation; indicator of intraoperative respiratory variability.
mean_abs_delta_etco2	Mean absolute change in end-tidal CO_2_; reflects ventilation and perfusion dynamics.
mean_abs_delta_rr	Mean absolute change in respiratory rate; ventilation pattern variability.
mean_abs_delta_vt	Mean absolute change in tidal volume; indicator of ventilation variability.
proc_HPRO_Hip_Prosthesis	Indicator variable for hip replacement or prosthesis-related procedures.
asa	American Society of Anesthesiologists (ASA) physical status classification.
max_art_dbp	Maximum intraoperative arterial diastolic blood pressure.

## Data Availability

The INSPIRE perioperative dataset analyzed in this study is available through PhysioNet (https://physionet.org/content/inspire/1.3/). The full implementation of the domain-aware interpretable machine learning model for predicting postoperative hospital length of stay from perioperative data, is openly available at www.github.com/iqram20/domain-aware-los.
